# Immunohistochemical expression of EGP40, a tumor marker, in different grades of oral squamous cell carcinoma

**DOI:** 10.4103/0973-029X.80038

**Published:** 2011

**Authors:** Vineeta Gupta, Nirmala N Rao

**Affiliations:** *Department of Oral Pathology and Microbiology, College of Dental Surgery, Manipal, India*

**Keywords:** EGP40, ESA, GA733-2, KSA, OSCC, 17-1A

## Abstract

**Aim:**

The aim of this study was to see the distribution and pattern of staining of epithelial glycoprotein 40 EGP-40 (also known as GA733-2, ESA, KSA, 17-1A antigen) in the different grades of OSCC.

**Materials and Methods:**

30 biopsy reports retrieved from the files of the Department of Oral Pathology and Microbiology, College of Dental Surgery, Manipal were used. These comprised of 10 microslides each of 3 different histological grades of Oral Squamous Cell Carcinoma, namely Well Differentiated, Moderately Differentiated and Poorly Differentiated carcinomas. Immunoperoxidase staining for IgG, was performed by the unlabelled antibody peroxidase-antiperoxidase complex (PAP) method. The criteria used to define an antigen positive area were: Homogenous /Patchy staining of the section and Cytoplasmic/ Membranous staining of the tumor cells.

**Results:**

The expression of EGP 40 in different grades of OSCC showed an inverse relationship to differentiation and a direct relationship with the proliferation of the tumor cells and its expression became more pronounced as the grade worsened i.e. from well to poorly differentiated OSCC.

**Conclusion:**

In the present study, the surface antigen EGP40 (Ep-CAM) was detected in the different grades of OSCC with its expression becoming more pronounced as the grade worsened i.e. from well to poorly differentiated OSCC. However, further studies are required to understand the dualistic role of EGP40 (Ep-CAM) in mediating cell to cell adhesion preventing cell scattering and its heterogeneous expression in promoting tumor invasion and metastasis and also to determine its exact role and significance at a practically applicable level.

## INTRODUCTION

In today’s pathology, many investigations are undertaken to determine the molecular mechanisms that are associated with progression from normal tissues, through various stages of dysplasia, to carcinoma *in situ* and ultimately to invasive squamous cell carcinomas. Normal epithelial cell surface contains a complex and functionally diverse array of membrane glycoproteins which mediate cell to cell interactions and cellular recognition. These often have been recognized through characterization of surface antigens on tumor cells by the development of monoclonal antibodies. Among these surface antigens defined by monoclonal antibodies (Mab), epithelial glycoprotein 40 (EGP40; a.k.a. GA733-2, ESA, KSA, 17-1A antigen) is expressed in vast majority of carcinomas and has attracted attention as a tumor marker.[1,2] Immunohistochemical studies on human tissues have demonstrated the expression of EGP40 in almost all normal epithelial tissues encoded by *GA733-2* gene, expressed at the lateral and sometimes on the basal domains of cell membranes.[3] As EGP40 also shares sequence homology to nidogen, an extracellular matrix protein, and to placental protein 12, it suggests that EGP40 may be a member of a tumor associated antigen family of adhesion molecules which are involved in cell-cell or cell-matrix interactions. A few data also suggest that EGP40 is functionally active in many tumors and is located at intercellular boundaries in many carcinomas, similar to its location in normal tissues.[3,4] In view of its adhesion function and its presence only in epithelial tissue, the name Ep-CAM is correlated with proliferation, decreased cadherin-mediated adhesion and differentiation of the tumor cells. In the present study, an attempt has been made to study the expression of EGP40 (Ep-CAM) on different grades of oral squamous cell carcinoma (OSCC) which may contribute towards immunopathological diagnosis, in the development of new therapeutic strategies by employing this antigen as a target.

## MATERIALS AND METHODS

A total of 30 biopsy reports retrieved from the files of the Department of Oral Pathology and Microbiology, College of Dental Surgery, Manipal, constituted the materials for the study. The 30 microslides were made up of 10 microslides each of three different histological grades of OSCC, namely well-differentiated, moderately differentiated and poorly differentiated carcinomas. All the tissues were fixed in neutral buffered formalin, and after routine processing, they were embedded in paraffin to form blocks. From these blocks, sections of 5 mm were cut and floated on water (48-50°C) and mounted on glass microslides coated with chrome alum gelatin as an adhesive. These slides were then placed on a slide warmer at 50°C for min 30 min. Immunoperoxidase staining for IgG was performed by the unlabeled antibody peroxidase-antiperoxidase complex (PAP) method. The positively stained areas gave a reddish brown color.

The criteria used to define antigen positive areas were:

1. homogeneous/patchy staining of the section; and
2. cytoplasmic/membranous staining of the tumor cells.

In all sections, the invasive areas were assessed.

### Scoring system used

Staining was assessed in two steps:

- Assessment for homogeneous and patchy staining in the given section
  This was done under 100× using a compound microscope.
  Patchy staining was given value of 1.
  Homogeneous staining was given value of 2.
- Assessment for cytoplasmic and cell membrane staining
  This was done under 400× using a compound microscope.
  Cytoplasmic staining was scored +.
  Membranous staining was scored ++
  Two observers assessed the same sections simultaneously to minimize any subjective error that may occur. To find agreement between the observers, Kappa statistics was used.

$$k\;=\;\frac{P\left(A\right)\;-\;P\left(E\right)}{1\;-\;P\left(E\right)}$$

P(A) = proportion of times the k raters agree

P(E) = proportion of time that we expect k raters to agree by chance.

All statistical analyses were done by using the chi-square test wherein *P*<0.01 was considered to be significant.

## RESULTS

After immunohistochemical staining was done, the positive areas showed immunoreactivity in the cellular cytoplasm, on the membrane and, in some cases, in both the areas. This staining was seen as orange-brown color in the cytoplasm and as brown color on the membrane.

**Figure 1:**
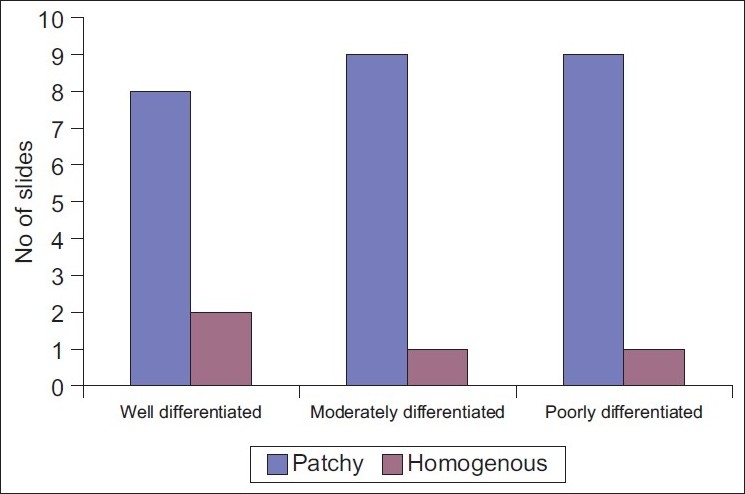
Demonstrating the pattern of (Homogenous/Patchy) expression of EGP40 in different grades of oral squamous cell carcinoma

**Figure 2:**
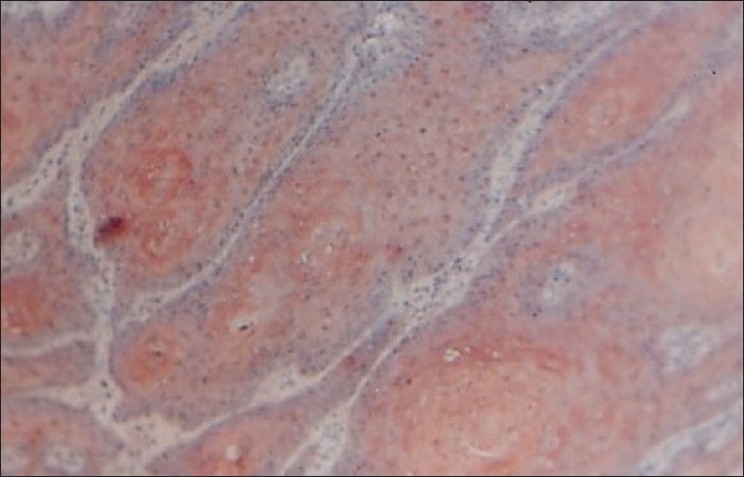
Photomicrograph shows the immunohistochemical distribution of EGP40 (AEC chromogen) in well-differentiated oral squamous cell carcinoma (homogeneous pattern of expression) 10×

**Figure 3:**
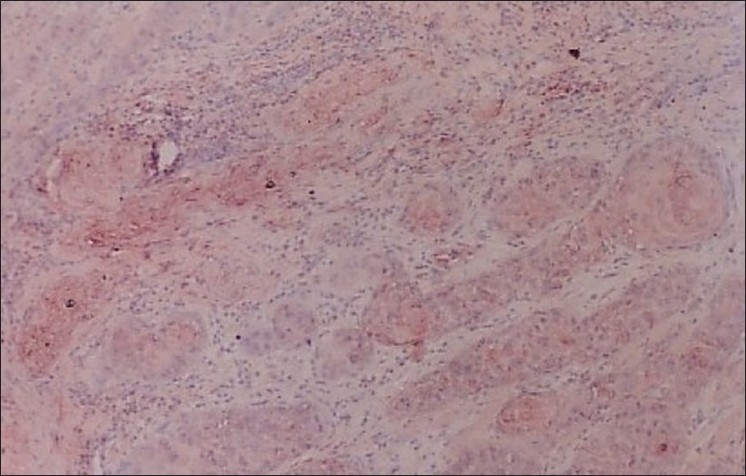
Photomicrograph shows the immunohistochemical distribution of EGP40 (AEC chromogen) in moderately differentiated oral squamous cell carcinoma (patchy pattern of expression) 10×

Homogeneous and patchy distribution was observed at a magnification of 100× [Table 1, Figures 1–3].

**Table 1:** Staining pattern of EGP40 in different grades of oral squamous cell carcinoma. (×100)

Grades	Patchy stain	Homogenous stain	Total	Test of significance results
Well differentiated (Grade I)	8	2	10	X^2^=0.577 *P*=0.749 NS
Moderately differentiated (Grade II)	9	1	10
Poorly differentiated (Grade III)	9	1	10
Total	26	4	30

**Figure 4:**
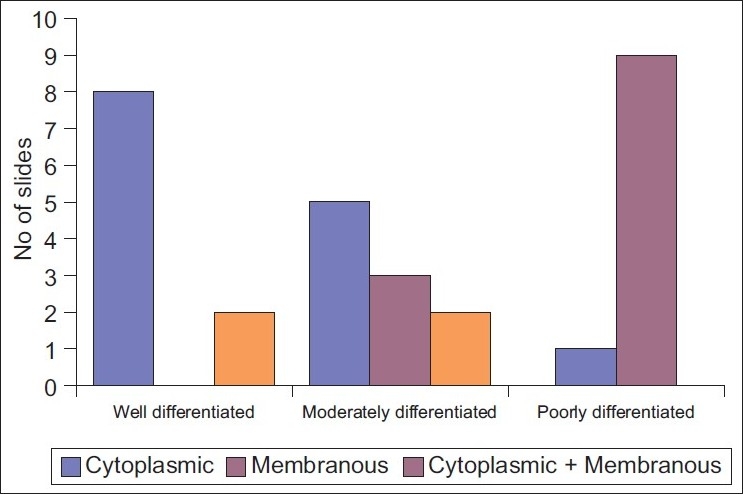
The distribution (Cytoplasmic/Membranous) of EGP40 in different grades of oral squamous cell carcinoma

**Figure 5:**
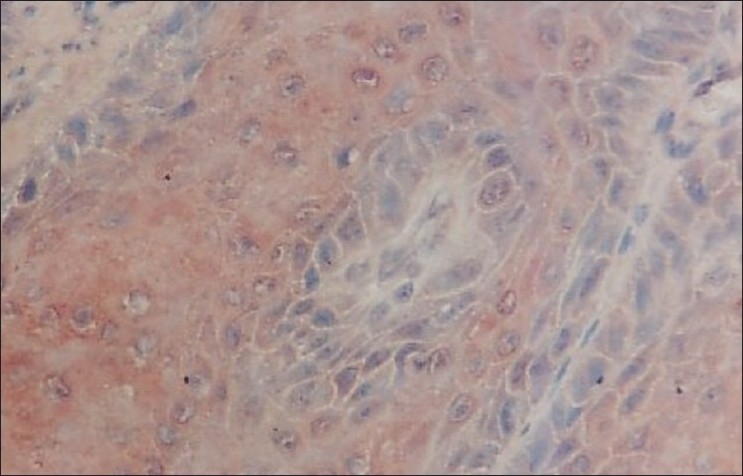
Photomicrograph shows the Immunohistochemical distribution of EGP40 (AEC chromogen) in well-differentiated oral squamous cell carcinoma (cytoplasmic distribution of expression) 40×

**Figure 6:**
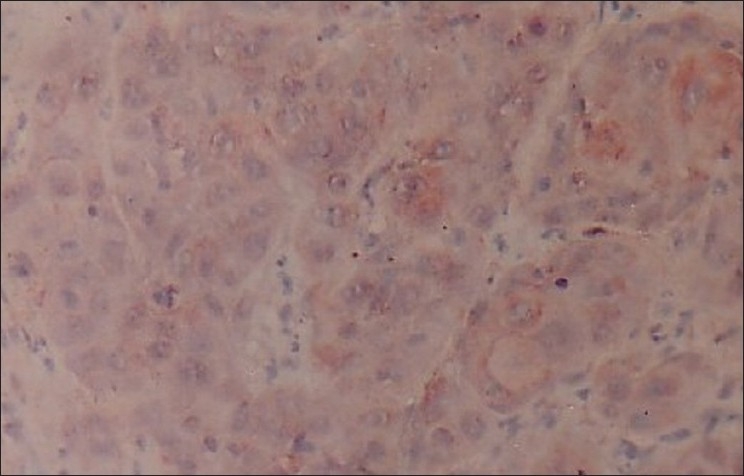
Photomicrograph shows the immunohistochemical distribution of EGP40 (AEC chromogen) in moderately differentiated oral squamous cell carcinoma (cytoplasmic and membranous distribution of expression) 40×

**Figure 7:**
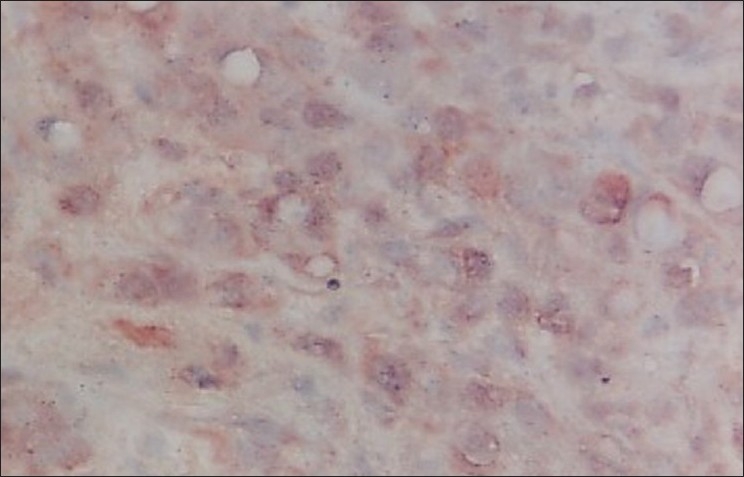
Photomicrograph shows the immunohistochemical expression of EGP40 (AEC chromogen) in poorly differentiated OSCC (membranous distribution) 40×

**Figure 8:**
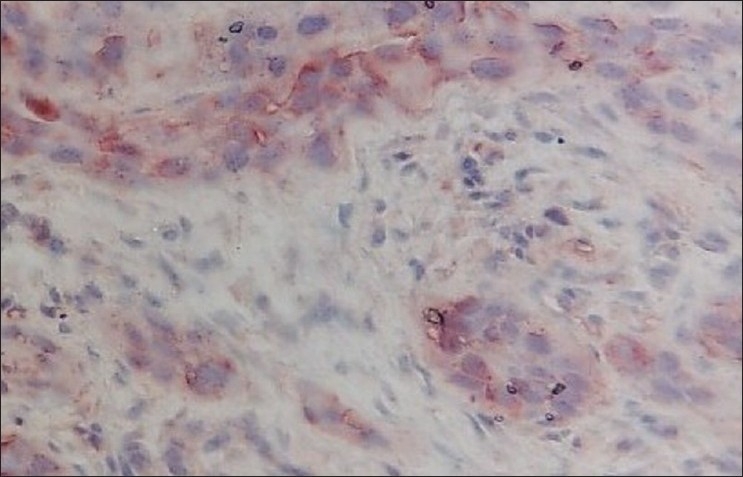
Photomicrograph shows the immunohistochemical expression of EGP40 (AEC chromogen) in poorly differentiated OSCC (membranous distribution) 40×

Cytoplasmic and membranous staining was observed at 400× magnification [Table 2, Figures 4–8].

**Table 2:** Distribution of EGP40 in different grades of oral squamous cell carcinoma. (×400)

Grades	Cytoplasmic stain	Membranous stain	Cytoplasmic + Membranous	Total
Well differentiated (Grade I)	8		2	10
Moderately differentiated (Grade II)	5	3	3	10
Poorly differentiated (Grade III)	1	9		10
Total	14	12	4	30

To find agreement between the observers, Kappa statistics was used. As k=0.82, a good agreement was seen between both the observers; so, only one of the two observer groups was considered in the analysis.

No statistical significance was noted between various grades of OSCC in relation to homogeneous or patchy staining (χ^2^ = 0.0577, *P*=0.749) [Table 1].

However, a very high statistical significance was observed between different grades of OSCC, in relation to cytoplasmic and membranous staining [Table 3].

In between well-differentiated and moderately differentiated squamous cell carcinoma, no statistical significance was observed (*P*>0.05). In between moderately differentiated and poorly differentiated squamous cell carcinoma, a high statistical significance was seen (*P*<0.01). A very high statistical significance was observed (*P*<0.001) between the well-differentiated and poorly differentiated grades [Table 3Figure 2].

**Table 3:** Inter-grade comparison of EGP40 staining in different grades of oral squamous cell carcinoma

Grades	*P* value	Significance
Well differentiated (Grade I) Vs	>0.05	Not significant
moderately differentiated (Grade II)		
Moderately differentiated (Grade II)	<0.01	Highly significant
Vs poorly differentiated (Grade III)		
Poorly differentiated (Grade III) Vs	<0.001	Very highly significant
well differentiated (Grade I)		

## DISCUSSION

Numerous attempts have been made during the last 20 years or so to define cell surface antigens of human cancer cells by generating monoclonal antibodies to assess the tumor cell biology, which may in turn form the basis of new modalities in the treatment of cancer. *In vitro* and *in vivo* studies have shown that in many human tumors, there is widespread deregulated expression of cell adhesion molecules and these molecules are not only important in adhesion but also important to transduce signals into cells that control morphological differentiation, gene expression and cell motility.[5,6] In the present study, 5-µm thick paraffin-embedded sections of different grades of OSCC were stained by immunoperoxidase technique using Mab EGP40 [Table 1]. The results of the study indicate the expression of cell surface antigen EGP40 in all grades of OSCC. Additionally, a different pattern of staining was observed in different areas of the same tumor, patchy/homogeneous staining and intense cytoplasmic/membranous staining [Tables 1 and 2].

In the present study, grade I and grade II OSCC showed intense cytoplasmic staining of EGP40 (Ep-CAM). This could be due to the interaction of the cell surface glycoprotein (extracellular domain) with intracellular cytoskeletal domain for cell adhesion.[7] Studies have also shown that any deletion in this cytoplasmic domain of EGP40 (Ep-CAM) molecule may result in weak or unstable adhesions and a decrease in cell aggregation[2] which may promote invasion and metastasis from the carcinoma or from the primary tumor.[5]

Active proliferation in epithelial tissues is associated with increased or *de novo* EGP40 (Ep-CAM) expression. This was especially evident in early stages of neoplasms of uterine cervix. A *de novo* expression was observed in areas with atypical undifferentiated cells of the squamous epithelium.[8] Whilst in the present study grade III OSCC exhibited an intense membranous staining of EGP40 (EpCAM), this expression can be heterogeneous, i.e. of other origin, probably by a shift in tumor cell differentiation to either mesenchymal or squamous cell phenotype, therefore being expressed only on undifferentiated cells.[2]

The expression of this glycoprotein in different grades of squamous cell carcinoma is inversely correlated with the differentiation; it appears restricted to very immature cells of germline phenotype induced by cellular transformation [Table 3].

## CONCLUSION

In the present study, the surface antigen EGP40 (Ep-CAM), a novel cell surface glycoprotein, was detected immunohistochemically in the different grades of OSCC. It showed a variable pattern of staining in the tumor, observed as patchy/homogeneous and intense cytoplasmic/membranous. Its expression in different grades of OSCC showed an inverse relationship with the proliferation of the tumor cells. Its expression became more pronounced as the grade worsened, i.e. from well-differentiated to poorly differentiated OSCC. However, further studies are required to understand the dualistic role of EGP40 (Ep-CAM) in mediating cell to cell adhesion preventing cell scattering and its heterogeneous expression in promoting tumor invasion and metastasis and also to determine its exact role and significance at a practically applicable level.
